# How to Cope with the Challenges of Medical Education? Stress, Depression, and Coping in Undergraduate Medical Students

**DOI:** 10.1007/s40596-020-01193-1

**Published:** 2020-02-20

**Authors:** Verena Steiner-Hofbauer, Anita Holzinger

**Affiliations:** Medical University Vienna/Medizinische Universität Wien, Vienna, Austria

**Keywords:** Stress, Depression, Medical students, Coping, Mental health

## Abstract

**Objectives:**

Up to 90% of medical students experience stress. Studies have observed a relationship between stress and depression. Coping strategies to deal with stress and depression are of great interest. This study aimed to evaluate the prevalence of stress and depression and the efficacy of coping strategies in undergraduate medical students.

**Methods:**

This survey was conducted with 589 second-year and sixth-year students in 2017 at the Medical University of Vienna. The questionnaire included a stress and coping questionnaire, depression screening, substance use questionnaire, and questionnaire concerning leisure time activities.

**Results:**

The coping strategies were included in a regression model to assess their predictive value for stress and depression screening scores. The most common stressor was performance pressure overload (92.1%). Overall, 52.4% of the participating students reached critical scores in the depression screening. Positive thinking and active coping were associated with lower stress scores. Positive thinking also was a protective factor against depressive symptoms. Less than 2% of all students reached high-risk values for substance use.

**Conclusion:**

Accessible counseling for students in need of psychological care should be provided. Different interventions of positive psychology showed a positive impact on depression screening scores.

Being a doctor is a stressful profession, but studying medicine can also be challenging. Several studies have focused on stress among medical students. In a review, Fares et al. [1] showed that 20.9 to 90% of students experienced stress. Dyrbye et al. [2] emphasize that medical students work more hours than students in other higher education courses. Besides these factors, the common stressors experienced by medical students are their high workload and the broadness of the academic syllabus, frequency of examinations, high expectations of themselves, little time for hobbies, social isolation, or competition with their peers [3–5]. The timetables of medical curricula are overloaded, which often induces pressure and stress. The medical curriculum at the Medical University of Vienna is an integrated curriculum with big written exams at the end of every year and 48 weeks of clinical training in the sixth year at the end of their studies. Medical school students report that they were often stressed in the first years of their studies due to the big examinations; however, final-year students often struggle with the step from simulated training sessions into real-world hospital work in their clinical training and the responsibility connected with working with real patients as well as the prospect of finishing medical school and starting their professional career. Additionally, medical students are confronted with topics like death and grief; therefore, they are more often affected by depression [2]. A German study revealed that health-related quality of life is reduced in medical students and depression is frequent [6]. Saravanan and Wilks [7] found a significant relationship between stress and depression. To deal with stress and prevent depression, effective coping is necessary [8]. A review by Kumar [9] suggests three levels of burnout prevention, including reducing stress and poor health through effective coping and promoting healthy behaviors.

Coping can be a mainly attitudinal process comprising different mental approaches. Common coping strategies are “positive thinking” like reframing, humor, or optimism; “social support” like seeking emotional and instrumental support from other people; and “turning to religion” like trusting higher powers to find comfort [10]. But coping can also include different actions and activities like “active coping,” which includes activities to prevent stress from arising, like planning ahead or a strategic working approach [10]. In addition, the literature suggests that lifestyle and leisure time activities can be used as a coping strategy. Cheung and Yip [11] found an influence of lifestyle factors on depression with physical activity being a key factor in mental health. Using relaxation techniques can influence various mental health problems like anxiety or depression [12, 13]. Pekmezovic and colleagues [14] showed that health-related quality of life (including mental health) increases with the frequency of physical activity in a sample of college students. Other activities diverting from stress and burden are creative tasks like playing music [1], crafting, outdoor activities, or meeting friends, sleeping, or consuming media [15]. We classify all the above-mentioned strategies as functional coping because their utilization entails no immediate risks.

However, the use of dysfunctional coping strategies, like drinking or consuming other legal or illicit drugs, is associated with various health risks and can only conceal stress or depression. Substance use among medical students and doctors was the subject of several studies and showed a high prevalence of substance use [16–18]. Legal or illicit drugs may be used to self-medicate depression or serve as an immediate distraction from stress. Harris et al. [4] and Park and Levenson [19] found that around 40% of students used alcohol as a coping strategy and highlighted that medical students are not invulnerable to excessive drinking, smoking, or abuse of illicit drugs [20]. Furthermore, substance use is not always reduced when students enter professional life as doctors [18]. Marshall [21] identified stress and depression, as well as the privileged access to mood-altering drugs, as risk factors for substance misuse in doctors.

To our knowledge, data on stress, depressive symptoms, and coping techniques of medical students in Austria are lacking. To fill this research gap, we conducted a cross-sectional study at the Medical University of Vienna. This study aims to identify:

- The prevalence of stress and symptoms of depression among medical students,
- The prevalence of the following functional and dysfunctional coping strategies: substance use, leisure time activities, active coping, positive thinking, faith/religion, and social support,
- The prevalence of substance use (of legal and illicit drugs) among students,
- If there is a difference in the above between male and female students,
- If there is a difference in the above between students in preclinical (second-year students) and clinical training (sixth-year students),
- The association between the measured coping strategies, and stress and critical depression screening scores.

## Method

To find out details about the mental health of students at the Medical University of Vienna, a cross-sectional survey was conducted among students during the preclinical training in the second-year and sixth-year students after their clinical training. This sampling strategy makes it possible to compare the preclinical level, mainly at the beginning of the study, and the clinical level after 1 year of clinical internship at the end of medical education.

In the second-year group, 424 out of 639 students participated (65.9%). The participants of the second-year group were aged between 19 and 26 years, with a mean age of 21.4 years, 47% were men, 53% were women, 89.4% were students of general medicine and 10.6% dentistry. In the sixth-year group, 165 out of 363 students participated (45.5%), 53.4% were men, 46.6% were women, 64.8% of the students were aged between 23 and 26 years, and 35.2% were 27 years or older. No dentistry students participated in the sixth-year group.

We distributed the paper-and-pencil questionnaires (including an envelope) during mandatory seminars and asked the students to put the completed questionnaire (in the sealed envelope) in provided collecting boxes placed in all seminar rooms. The collection was completely anonymous and voluntary. All participants gave informed consent. The data protection commission and the internal ethics committee of the Medical University of Vienna approved this study.

### Questionnaire

We created a questionnaire consisting of several standardized questionnaires and self-developed questions regarding leisure time activities, relaxation techniques, and demographic data: age, gender, and country of birth. The following standardized and self-developed questionnaires were used:

#### Stress and Coping Inventory

The Stress and Coping Inventory (SCI) [10] includes questions about burdens, perceived overextension, and uncertainties as well as symptoms of stress and pressure and coping strategies. Burdens, perceived overextension, and uncertainties are rated on a 7-point Likert scale and summarized to a total stress-score. Coping strategies are measured on a 4-point Likert scale and include active coping, coping through positive thinking, coping through faith or religion, and coping through substance use.

#### Center for Epidemiologic Studies Depression Scale

The Center for Epidemiologic Studies Depression Scale (CES-D) [22] is a depression-screening questionnaire consisting of 20 items. It measures symptoms defined by the American Psychiatric Associations Diagnostic and Statistical Manual (DSM-5) for a major depressive episode. Contents are sadness, loss of interest, appetite, sleep, thinking and concentration, guilt (worthlessness), tiredness (fatigue), movement (agitation), and suicidal ideation in the last 7 days on a 4-point scale. All values below 16, across all 20 questions, are of no clinical significance.

#### Alcohol, Smoking and Substance Involvement Screening Test

The ASSIST, Alcohol, Smoking and Substance Involvement Screening V 3.0 Test was developed by the WHO to detect substance abuse and related problems [23]. It includes questions about the following substances: tobacco, alcohol, cannabis, cocaine, amphetamines, inhalants, sedatives or sleeping pills, hallucinogens, opioids, and others. For this study, neuro-enhancement drugs were added to the list. The ASSIST questionnaire can determine a risk score for each substance. Maximum range is 0–33. Low risk means 0–3 points for all substances except alcohol; for alcohol 0–10 points are low-risk scores. Medium risk means 4–26 points for all substances except alcohol; for alcohol 11–26 points are medium-risk scores. High risk means ≥ 27 points for all substances. For “substance use” as a dysfunctional coping measure, we used the reported consumption of all substances during the last 3 months.

#### Leisure Time Activities

Leisure time activities included activities drawn from literature and common relaxation techniques. The questionnaire included sports, sauna, or wellness, meeting friends, cooking, reading, watching films or tv shows, listening to music, being creative (activities such as music, painting, crafting), outdoor activities, sleeping, partying, others as well as the use of relaxation techniques (yoga, tai chi, progressive muscle relaxation, autogenic training, meditation, and sports for relaxation). An activity performed at least one or two times a week was considered a hobby and multiple answers were possible (1 = hobby, 0 = not a hobby). Additionally, these activities could be chosen as a coping strategy. Relaxation techniques were rated as 0 = never practiced, 1 = practiced when needed, and 2 = practiced on regular basis. The sum of scores was used as a reference for versatility of private life and anticipated as functional coping. The range of points was 0 to 40. This instrument was developed by the authors based on the HISBUS survey [15] for the German Ministry of Health.

### Statistical Analysis

For data analysis, SPSS software (v. 24.0.0.0; SPSS IBM, Armonk, NY, USA) was used. We calculated correlations, regression analysis, and an ANOVA for score differences and a regression analysis for associations between stress, critical depression screening scores, and coping. Little’s test was conducted to test missing values for missing completely at random (MCAR). MCAR assumption was met. Due to our large sample, list-wise deletion was chosen for handling the missing data.

## Results

Overall stress scores were 50.7 points in the sixth year and 44.3 points in the second year with a mean of 47.5 over both groups. In both years, women experienced significantly more stress than men did (*p* < .05; second year, 49.2 vs. 43.8 points in SCI scores; sixth year, 55.3 vs. 45.5 points in SCI scores). Table 1 shows the top five stressors for second- and sixth-year female and male students (Table 1).

**Table 1:** Top 5 stressors of female and male students of 2nd and 6th year (%)

Female	%	Male	%
2nd year		2nd year	
Performance pressure overload	92.2	Performance pressure overload	88.3
High expectations for themselves	91.3	High expectations for themselves	81.1
Uncertainty about life goals	72.5	Uncertainty about life goals	69.4
Uncertainty about studies	67.9	Uncertainty about studies	59.2
Social expectations	60.1	Uncertainty about relationship	54.6
6th year		6th year	
Performance pressure overload	85.5	Uncertainty about studies	80.5
High expectations for themselves	85.5	High expectations for themselves	76.8
Uncertainty about studies	84.2	Uncertainty about life goals	74.7
Uncertainty about life goals	82.9	Performance pressure overload	73.6
Financial uncertainties	77.6	Financial uncertainties and uncertainty about relationship	56,3

Overall, 52.4% of the participating students had critical values of ≥ 16 in their CES-D depression screening. More than half (59%) of the participating second-year students had critical values of ≥ 16 in the CES-D depression screening. Of all female second-year students, 68.5% had critical values in the CES-D screening with a mean score of 22.8 (SD, 11.5) as well as 48.4% of all male students with a mean score of 16.6 (SD, 9.8). In the sixth-year group, 35.2% of the participating students had a score of ≥ 16, with 37.1% of female and 33.3% of male students affected. The mean scores of the sixth-year students were the following: women, 14.6 (SD, 7.7); men, 13.9 (SD, 10.4). The gender differences reach statistical significance in the second year and overall (*p* < .05). Stress and depression screening scores were significantly correlated (*p* < .05).

Table 2 shows the coping strategies of female and male second- and sixth-year students and overall. Leisure time activities showed a moderately versatile private life in all groups. Substance use was low. Social support was the most popular coping strategy (Table 2).

**Table 2:** Coping strategies in female and male students of 2nd and 6th year and overall.

Coping Strategy	Female Mean (SD)	Male Mean (SD)	Range
2nd year			
Positive thinking	10.7 (2.5)	11.0 (2.3)	4–16
Active coping	10.3 (2.6)	10.2 (2.6)	4–16
Faith/religion	7.4 (2.8)	7.0 (2.8)	4–16
Social support*	12.4 (2.0)	11.7 (1.9)	4–16
Leisure time activities	12.0 (3.8)	10.8 (3.4)	0–40
Substance use*	4.9 (3.7)	6.1 (5.6)	0–66
6th year			
Positive thinking	10.7 (2.2)	11.1 (2.4)	4–16
Active coping	10.0 (2.5)	10.4 (2.7)	4–16
Faith/religion	7.1 (2.7)	6.8 (2.4)	4–16
Social support*	14.3 (2.2)	13.2 (2.9)	4–16
Leisure time activities	11.4 (4.1)	10.8 (4.2)	0–40
Substance use	4.2 (2.7)	5.0 (3.0)	0–66
Overall			
Positive thinking	10.7 (2.4)	11.0 (2.3)	4–16
Active coping	10.2 (2.6)	10.3 (2.6)	4–16
Faith/religion	7.3 (2.8)	6.9 (2.7)	4–16
Social support*	12.9 (2.2)	12.1 (2.4)	4–16
Leisure time activities*	11.8 (3.9)	10.8 (3.6)	0–40
Substance use*	4.7 (3.5)	5.77 (5.0)	0–66

Higher values indicate a more frequent use of the strategy. Standard deviations in parentheses. Asterisks indicate significant gender differences (*p* < .05). Range shows the possible range of the respective variable

The 3-month prevalence of substance use and percentage of participants with high- or low-risk scores in female and male second- and sixth-year students is shown in Table 3. Alcohol was the substance with the highest prevalence in all groups, followed by tobacco and cannabis. The use of neuro-enhancements, amphetamines, opioids, and cocaine was rare. High-risk substance use was also rare (Table 3).

**Table 3:** Three-month prevalence of substance use and percentage of participants with high- or low-risk scores in female and male students of 2nd and 6th year

Substance	Female			Male		
	3-month prevalence %	Risk score		3-month prevalence %	Risk score	
		Low %	High %		Low %	High %
2nd year						
Alcohol	90.2	16.5	0.5	93.2	27	0
Tobacco	32.2	25.2	1	38.2	26	1.6
Cannabis	21.0	17.4	0	34.0	22	1.0
Neuro-enhancement	0.9	4.5	0	2.1	1.8	0.6
Sedatives/sleeping	5.1	4.0	0	4.8	3.4	0.6
Amphetamines	3.8	1.5	0	4.7	6.3	0
Opioids	0.9	0	0	3.1	3.4	0.6
Cocaine	0.5	0.5	0	3.6	3.2	0.6
6th year						
Alcohol	91.7	11.2	1.4	93.7	22.7	0
Tobacco	19.4	18.5	1.4	26.6	26.5	0
Cannabis	15.3	8.4	0	21.5	11	0
Neuro-enhancement	1.4	1.4	0	1.9	2.8	0
Sedatives/sleeping	2.8	1.4	0	0	0	0
Amphetamines	0	0	0	1.3	1.3	0
Opioids	0	0	0	0	0	0
Cocaine	0	0	0	1.3	1.3	0

Low risk means 0–3 points for all substances except alcohol, for alcohol 0–10 points are low-risk scores. High risk means 27 points and more for all substances. Maximum range is 0–33

A linear regression was calculated to predict participants’ stress and depression screening scores based on the utilization of different coping strategies: i.e., positive thinking, social support, religion/faith, leisure time activities, and substance use. Participants’ stress scores decreased 1.6 points for each point in positive thinking (*β* = − 0.211, *p* = 0.000**), decreased 0.9 points (*β* = − 0.140, *p* = 0.006**) for each point in active coping and increased 0.7 points (*β* = 0.111, *p* = 0.026*) for each point in coping through faith, with an *R*^2^ of 0.089. Depression screening scores were significantly associated only with positive thinking. Participants’ depression screening scores decreased 1.9 points (*β* = − 0.401, *p* = 0.000**) for each point in positive thinking, with an *R*^2^ of 0.185. All other associations were not significant.

## Discussion

In this study, we investigated the prevalence of stress and depressive symptoms as well as the prevalence of functional and dysfunctional coping strategies among male and female students in clinical and preclinical training. However, the authors wanted to know if and how stress and depression screening scores are associated with different coping strategies. Second-year students experienced more stress than their sixth-year colleagues. Female students in both groups experienced more stress than did male students. Critical depression screening scores of ≥ 16 were more prevalent in the second-year group. Overall, about 50% of all students obtained critical depression screening scores in the CES-D screening. Seeking social support was the most frequently used coping strategy, but it reduced neither stress nor depression screening scores. Turning to religion was slightly associated with higher stress scores. Active coping and positive thinking were associated with lower stress scores and positive thinking was associated with lower depression screening scores. Risky substance use was uncommon in our sample. Alcohol, tobacco, and cannabis were the most used substances; the use of other drugs was rare.

In their systematic review of 195 studies, Rotenstein et al. [24] found an overall prevalence of depression or depressive symptoms of 27.2% with a wide range between 1.4% and 73.5%. This indicates a higher prevalence of depression in our group than in the general public [25]. Many factors can influence the occurrence of high depression scores. A detailed examination of the review data of Rotenstein et al. [24] showed that the mean prevalence of depression in studies that used the CES-D was 43.3% and therefore it was higher than the overall prevalence. However, many of the students, especially in the second year, had high scores in the CES-D depression screening, which correlated significantly with high stress scores.

In our study, the highest-rated stressors were performance pressure and the high standards that the students set for themselves. In both groups, female students experienced more stress than their male colleagues did, but only in the second-year group this higher stress scores occurred combined with higher scores in the depression screening. The connection between stress and depression is not exhaustingly explained. Frequent stress does not necessarily lead to depression, but a frequent negative perception of stress can be harmful [26]. Zong et al. [27] showed the importance of empowering undergraduate students with adaptive skills to cope with a stressful life because depression can lead to increased utilization of maladaptive (dysfunctional) coping methods.

A versatile private life including sport and social support showed no significant connection to stress or depressive symptoms in our study. Even though social support has proven to be a strong coping strategy to deal with stress and burden in several studies, Baqutayan [28] showed that only social support based on counseling lessons had a significant positive effect on stress in young students. Support from friends and family showed no significant effects. Lee and Goldstein [29] found that support from friends and romantic partners can buffer negative effects of stress in young adults but not family support. Dyrbye et al. discovered a similar effect between social support and burn out in medical students. Their results suggest that social support, especially from faculty members and in the form of a positive learning climate, is important [30]. Haldorson et al. [26] could not find a clear connection between social support and depression. They also concluded that according to Cohen et al., social support can only reduce depressive symptoms when it mirrors the exact needs of the recipient. We did not investigate the kind of social support in detail in our study. For future research, a focus on different aspects of social support might be fruitful. A detailed analysis of leisure time activities showed almost no difference between the groups of students with critical and noncritical depression screening scores. Wolf and Rosenstock [31] also failed to establish the significant influence of sleeping habits and exercising on depression. Despite this fact and due to mixed results in literature, as well as for the manifold health benefits of regular sleep and exercising habits, they recommend interventions to improve healthy sleeping and exercising habits. A study by Zanardelli and colleagues [32] examined functional coping strategies, but failed to show any association with well-being. They assumed that functional coping strategies only maintain a baseline level of functioning, but may not improve the levels of perceived well-being. This assumption would underpin our findings that most strategies do not have effects on depression screening scores and only minor effects on stress.

In our study, only positive thinking was associated with lower depression screening scores. Even though Bolier et al. [33] found that interventions that enhance positive thinking skills could enhance well-being and reduce depression scores, it is questionable if positive thinking can be used as a coping strategy to deal with an already existing depression because one of the main symptoms of depression is the impaired capability to think positively. The authors understand the positive capacity of positive thinking more in a preventative way. If a person is capable of positive thinking or uses positive psychology interventions to improve their positive thinking skills, this may serve as a protective factor preventing depression from arising.

Religious coping was significantly associated with higher stress scores in our sample and showed no association with depression screening scores. Terreri and Glenwick [34] suggested that even though believing in higher forces can be helpful in some situations, having an “external locus of control” could be the reason that religious coping has a negative effect on stress. Additionally, they argue that negative religious coping (feeling abandoned or punished by God) can lead to increased feelings of helplessness and powerlessness. According to Pargament [35], people primarily find meaning in significant life events through religion and do not find the same help for their daily hassles. In both of our samples, substance use could predict neither depression screening scores nor stress. While Erschens et al. [36] emphasize that substance use increases the risk of burnout and Zanardelli and colleagues [32] reported that substance use was associated with lower well-being, Pickard et al. [16] found no correlation between substance use and anxiety and depression. He interpreted this as being positive because the students were not using drugs as a coping strategy. In contrast to other studies, which report about 30% of risky drinking habits [37] and alcohol abuse or dependence [38] among medical students, our data showed that even if the 3-month prevalence of alcohol consumption is over 90%, high-risk drinking was not very common. Overall, under 1.5% of our participants met the criteria for high-risk drinking. It can be assumed that substance use was not a popular way to reduce stress or self-medicate depressive symptoms in our sample. Research indicates that harmful substance use behaviors may occur well before students enter medical school [37, 39]. In Austria, a difficult and sophisticated admission test is in use to assign the available places at university. In 2018, only 13% of all applicants could acquire a place [40]. Predictive behaviors for substance use may influence the test outcome and a predisposition of ambition, eagerness, and determination paired with a strict schedule may lead to a reduced risk of substance abuse.

The cross-sectional design and that we used only data from one medical university are two major limitations of this study. Social desirability, disbelieving in the anonymity of the survey, and the fear that honest answers could have negative effects on their careers as well as voluntary participation can lead to systematically skewed data. The group of sixth-year students was notably smaller than the second-year group; therefore, statistical analysis between the groups may be impaired.

The low substance use rates and frequent use of functional coping leave us with a student population that has mainly problems with depressive symptoms. To support students with high levels of stress and depressive symptoms, it might be valuable to offer guidance through the process of reflecting on their career choices, standards, and expectations for themselves and facilitate a positive outlook on life and education. Different approaches could be tried because there is no sure formula for reducing stress and preventing depression in students. Heinzlmaier [41] highlights the importance of self-reflection to reduce exhaustion and depression among young people. Whitmore et al. [42] reported the positive effect of a reflective writing curriculum on medical students’ reflective skills. Positive psychology interventions to improve positive thinking skills may also be a way of enhancing the students’ well-being and prevent depressive symptoms [33]. Wild et al. [13] showed the effectiveness of an elective “relaxation techniques” course where students learned autogenic training and progressive muscle relaxation to conquer their stress. Medical schools should also offer easily accessible counseling for students in need of psychological care or support [32]. Additionally, universities should feel obliged to assess their curricula for especially stressful parts and try to implement necessary changes. Selection strategies could include assessments of resilience for mental health problems or identifying at-risk groups like “Type A” personalities [43]. Changing learning environments, as Dyrbye et al. [30] suggest, could also be a key element for improvement. Student mental health and well-being is a complex topic with many variables that must be considered by the stakeholders. If many connections remain unclear, a multidimensional approach could be appropriate and fruitful.
